# Supplementary material for: Learning curve and surgical outcome of robotic assisted colorectal surgery with ERAS program
Source: Sci Rep. 2022 Nov 29;12:20566. doi: 10.1038/s41598-022-24665-w (PMC9709162; doi:10.1038/s41598-022-24665-w)
Supplement: Supplementary file 2 — Supplementary Information 2. [file 41598_2022_24665_MOESM2_ESM.zip › RAL Raw data-Ñ[▒K/105/OP_ñw╝╨░O▒Kñσ.pdf]

正式摘要(電子簽章：000000223261972)

請選擇...reporter

臺中榮民總醫院

Operation Note

[redacted]

- 手術日期：2021/08/18
- 手術醫師：VS 林俊余，CR 楊曙榮，R 洪浚嚴
- Anesthesia : GE
- Position : Supine
- Aseptic agents : Soap , Betadine Alcohol , Betadine Aqua

Diagnosis

- Pre-operative diagnosis : appendix tumor
- Post-operative diagnosis : Ditto

Surgical Indication

- Tumor resection

Operation

- Robot Assisted Right Hemicolectomy

Finding

- 1. One 2x4 cm tumor located at cecum
  2. There was no palpable liver metastasis, no visible peritoneal seeding.
  3. intra corporal anastomosios with Stapler 45 isocolic manner and close with handsew methods.
  4. Drain: 12 penrose in subhepatic space, Hyalobarrier+
  5. ICG: colon: 35sec. Anastomosis: 32 sec

Procedure

- Under GE, the patient was put in supine position. 5 troca hole was made, the first was by open method. Pneumoperitonium was created. the robotic endoscope was inserted. Abdominal exploration was done and the findings were mentioned above. The patient position was adjusted to head-down and left tilting to let small bowels move to LUQ and the A-colon was well exposured. The ileocolic vessel was identified, dissected and transected by hemolock. The retroperitoneal dissection was done through embryonic plane between meso-right colon and Gerota fascia. The Duodenum and pancrease head was identified and protected. The mobilization of A-colon was done. Intracoporeal resection of ileum and A-colon with side to side anastomosis was done by endo GIA 45mm. Tha ICG test was done and good blood supply noted. The lower abdoman incision was made and the A-colon was removed.
  The penrosce drain was placed to the subhepatic space. 3 ports and wound were closed in layers. Throughout the whole course of operation, patient stood quite well. The patient was sent to POR for post OP care.
- Estimated blood loss: 100 c.c
- 器械數量: 正確
- 紗布數量: 正確

Image

- PAINT
  - abdomen

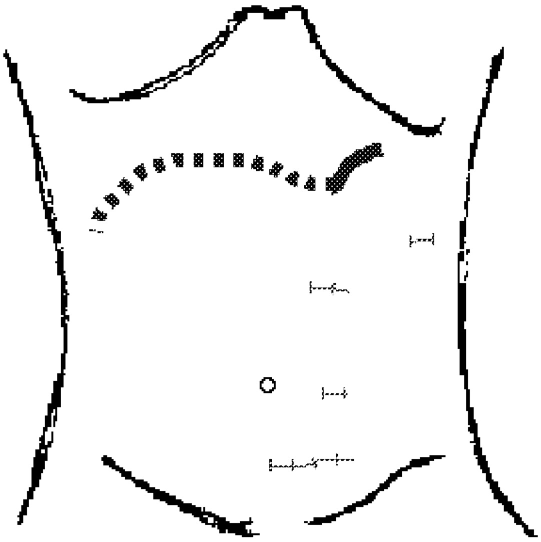

- colon & rectum

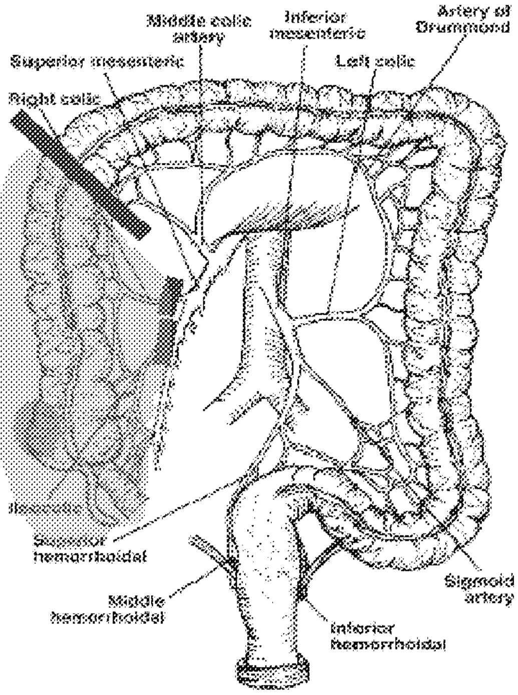

主治 2222H林俊余 / 醫師 2292G楊曙榮

正式摘要**(電子簽章：000000223261972)**

		請選擇...reporter			

doctorRank:B
